# Risk factors of multidrug-resistant bacteria in community-acquired urinary tract infections

**DOI:** 10.4314/ahs.v21i1.28

**Published:** 2021-03

**Authors:** Ertugrul Guclu, Fikret Halis, Elif Kose, Aziz Ogutlu, Oğuz Karabay

**Affiliations:** 1 Department of Infectious Diseases and Clinical Microbiology, Sakarya University Faculty of Medicine, Sakarya, Turkey; 2 Department of Urology, Sakarya University Faculty of Medicine, Sakarya, Turkey; 3 Department of Public Health, Sakarya University Faculty of Medicine, Sakarya, Turkey

**Keywords:** Urinary tract infection, community acquired, multidrug-resistant, male, multiple antibiotic usage, advanced age

## Abstract

**Background:**

Urinary tract infections (UTIs) are one of the most seen infection among community.

**Objectives:**

In this cross-sectional study we aimed to investigate the risk factors of multidrug-resistant (MDR) bacteria that caused community-acquired UTI (CA-UTI).

**Methods:**

Consecutive patients admitted to the Urology and Infectious Diseases policlinics with the diagnosis of CA-UTI were included in the study. A standard form including possible predisposing factors for MDR bacteria was applied.

**Results:**

In total, 240 patients (51.3% females) were enrolled in the study. The mean age of participants were 59.8 ± 18.3 years old. *Escherichia coli* (n =166; 69.2%)was the most frequently isolated bacteria and its incidence was higher in females than in males (p=0.01). In total, 129 (53.8%) of the identified pathogens were MDR bacteria. According to multivariate analysis, the use of antibiotics three or more times increased the risk of infection with MDR bacteria by 4.6 times, the history of urinary tract infection in the last 6 months by 2 times, being male and over 65 years old by 3 times.

**Conclusion:**

Doctors should consider prescribing broad-spectrum antibiotics in patients with severe UTIs with a history of UTI, advanced age, male gender, and multiple antibiotic usage, even if they have a CA-UTI.

## Introduction

Urinary tract infections (UTIs) are common infectious diseases in the community. Clinical manifestations of UTIs range from asymptomatic bacteriuria to serious diseases such as urosepsis and septic shock.^1^ The incidence of UTI is much higher in females during adolescence and childbearing years. Adult women develop approximately 30 times more urinary tract infections than men. After the age of 60, the frequency of UTI in men approaches women.^2^ The prevalence in females older than 65 years of age is approximately 20% and approximately 11% of the overall population.^3^ Other risk factors for UTIs in the community setting are the previous history of UTI, sexual activity within the past 48 h, pregnancy, neurogenic bladder dysfunction, and diabetes mellitus.^4^,^5^

UTIs are caused by a wide range of pathogens, including Gram-negative and Gram-positive bacteria, and fungi. Uropathogenic *Escherichia coli* is the dominant infectious agent in both uncomplicated and complicated UTIs with a prevalence of 75% and 65%, respectively. *Klebsiella pneumonia, Proteus mirabilis, Pseudomonas aeruginosa*, and *Enterobacter spp.* are other common Gram-negative uropathogens.^6^ Approximately, 15% of cases were due to Gram-positive pathogens such as *Staphylococcus saprophyticus, Enterococcus spp.*, and Group B *Streptococcus*.^7^ There is an increasing tendency of antimicrobial resistance in urinary pathogens. In the past, these resistant microorganisms were mainly detected in comorbidities such as diabetes mellitus, reflux nephropathy or nosocomial infections. However, they are found in an important part of community-acquired UTIs (CA-UTIs) in nowadays. This trend is not limited to any country but a global problem and has been reported to be seen in all groups.^8^ Advanced ag, the previous stay in care residence, prolonged hospital stay, presence of invasive devices such as urinary catheter, endotracheal tube, nasogastric tube, arterial pathways, gastrostomy or jejunostomy, administration of parenteral nutrition, recent surgery, hemodialysis, and poor nutritional status have been defined as the risk factors of the extended-spectrum beta-lactamase-producing pathogens. The use of antibiotics in previous months has also been identified as a possible risk factor, too.^9^

Risk factors for UTIs and multidrug-resistant (MDR) bacteria associated hospital-acquired UTIs have been well defined. However, risk factors for CA-UTIs due to MDR bacteria have not been identified clearly. This study aimed to investigate the risk factors that cause drug resistance in Gram-negative bacterial strains detected in CA-UTIs.

## Materials and methods

### Study design and setting

This study is a cross-sectional study in adults who applied to the Urology and Infectious Diseases Polyclinics of Sakarya University Training and Research Hospital between 01 January 2017 and 30 June 2019 with signs of UTI. All consecutive patients diagnosed with UTI were included in the study. Participants were asked to provide an on-the-spot urine sample for the investigation to determine true UTI cases. Patients were trained on how to collect mid-stream (clean-catch) urine samples according to standard methods. The urine samples were processed at the Medical Microbiology laboratory. A standard form was designed to record medical history information and filled after taking urine culture results.

### Survey

To detect predisposing factors for MDR bacteria, a standard form including possible risk factors was applied by face to face interview method. In the survey, in addition to the demographic data such as age and gender, the history of antibiotic use, hospitalization, urinary catheter insertion, surgery, and receiving immunosuppressive therapy in the last three months, presence of a healthcare worker in the family and comorbid diseases such as diabetes mellitus, renal failure, and hemodialysis were asked. Also, whether a person in his/her family has hospitalized or she/he has accompanied a hospitalized relative in the last three months were asked. The purpose of asking this question is to eliminate the risk of colonization with the hospital flora during the patient's stay as a companion. In antibiotic use patients, the number of times they used antibiotics and which antibiotics they use were questioned, too.

### Definitions

Urinary tract infection was defined as >10^5^ CFU /ml bacteria growth in urine culture in patients with lower urinary tract symptoms (dysuria, frequency, urgency, and suprapubic pain) or upper urinary tract clinical findings (costovertebral angle tenderness, fevers, urgency, dysuria, chills, nausea, and vomiting).

occured in the community or within 48 hours of admission to the hospital.

**Multidrug-resistant bacteria (MDR):** MDR was defined as acquired non-susceptibility to at least one agent in three or more antimicrobial categories.^10^ Extended-spectrum beta-lactamase-producing bacteria were defined as MDR, too.

**Inclusion criteria:** Patients diagnosed with CA-UTI who have Gram-negative bacteria in urine culture.

**Exclusion criteria:** Patients with the following criteria were excluded from the study.

- < 18 years old
- Nursing home patients
- Those with a history of hospitalization within the last 30 days.
- Patients with a history of antibiotic use in the last 30 days.
- Patients with urinary catheters in the last 48 hours or those use intermittent catheters.
- Patients with Double J ureteral stent.
- Patients admitted to the health facility for day-time treatment, hemodialysis or intravenous treatment within the last 30 days
- Patients who grow Gram-positive bacteria in urine culture.

### Statistical analysis

Statistical analysis was performed with SPSS version 20 software. Chi-square, fisher, Mann Whitney u tests were used during the univariate analysis of resistance development. Interactions and confounding factors were examined. Among the univariate analyses, p values of 0.25 and less were included in the model. In multivariate analysis, possible predictors of resistance development, independent predictors and impact modifiers were examined using logistic regression analysis. Hoshmer Lemeshow test was used for model fit. A 5% type I error level was used to infer statistical significance.

## Results

A total of 240 patients were enrolled in the study. Of these, 123 (51.3%) were females and 117 (48.7%) were males. The mean age of the participants was 59,8 ± 18,3 years old. Their age ranged from 18 years to 103 years. Of these, 106 (44.2%) patients (37 female and 69 male) were ≥ 65 years old and the number of males in this age group was higher than that of females (p = 0.000). The most frequently isolated species were Escherichia coli (n =166; 69.2%), *Klebsiella spp.* (n = 36; 15%), and *P. aeruginosa* (n= 11; 4.6%). The other isolated bacteria were *Enterobacter spp.* (n = 9; 3.8%), *Proteus spp.* (n = 8; 3.3%), *Serratia spp.* (n = 4; 1.7%), *Citrobacter spp.* (n = 3; 1.3%), *Burkholderia spp.* (n = 1; 0.4%), *Acinetobacter spp.* (n = 1; 0.4%), and *Providencia spp.* (n = 1; 0.4%). Ecoli was mainly responsible for UTIs in females (n = 94; 76.4 %), followed by *Klebsiella spp.* (n = 15; 12.2 %) and *Pseudomonas spp.* (n = 6; 4.9 %). Among males, 72 (61.5%) and 21 (17.9%) of isolated bacteria were *E. coli* and *Klebsiella spp.* The incidence of *E. coli* was statistically higher in females than in males (p=0.01). A higher proportion of *E. coli* was observed in females (81.1 %) compared to males (56.5 %) in patients aged over 65 years, too (p = 0.01). The distribution of isolated bacteria by gender is given in Table 1.

**Table 1:** The distribution of bacteria by gender

Bacteria	Male n = 117 (%)	Female n = 123 (%)	P value
***Escherichia coli***	72 (61.5)	94 (76.4)	0.01
***Klebsiella spp.***	21 (17.9)	15 (12.2)	0.21
***Pseudomonas aeruginosa***	5 (4.3)	6 (4.9)	0.82
***Enterobacter spp.***	5 (4.3)	4 (3.3)	0.93
***Proteus spp.***	6 (5.1)	2 (1.6)	0.24
**Others (*Serratia spp., Citrobacter spp., Burkholderi*** ***a spp., Acinetobacter spp., Providencia spp.)***	9 (7.7)	2 (1.6)	0.05

In total, 129 (53.8%) of the identified pathogens were MDR bacteria. While 69 (53.5%) of the bacteria detected in males were MDR, 60 (46.5%) of them in females were MDR (p = 0.14). Having urinary tract infection in the last 6 months was found risk factor for MDR (p < 0.05) (Table 2).

**Table 2:** Risk factors of patients for MDR bacteria related urinary tract infections according to characteristics of patients

Characteristics of patients	Non-multidrug resistant group n = 111 (%*)	Multidrug resis tant group n = 129 (%*)	P value
**Gender** **Male** **Female**	48 (43.2) 63 (56.8)	69 (53.5) 60 (46.5)	0.113
**Age, ≥ 65 years old** **Yes** **No**	45 (40.5) 66 (59.5)	61 (47.3) 68 (52.7)	0.29
**History of Urinary tract infection in the** **last 6 months** **Yes** **No**	33 (29.7) 78 (70.3)	67 (51.9) 62 (48.1)	**0.001**
**History of antibiotic use in the last 3 mo** **nths** **Yes** **No**	61 (55) 50 (45)	86 (66.7) 43 (33.3)	0.063
**History of urinary catheterization** **Yes** **No**	30 (27.0) 81 (73.0)	50 (38.8) 79 (61.2)	0.055
**Malignancy** **Yes** **No**	8 (7.2) 103 (92.8)	18 (14) 111 (86)	0.094
**Diabetes mellitus** **Yes** **No**	24 (21.6) 87 (78.4)	30 (23.3) 99 (76.7)	0.762
**Chronic renal failure** **Yes** **No**	9 102	12 117	0.74
**surgical operation in the last 3 months** **Yes** **No**	12 (10.8) 99 (89.2)	25 (19.4) 104 (80.6)	0,067
**Immunsupressive therapy** **Yes** **No**	2 109	9 120	0.10
**Hospitalizatin in the last 3 months** **Yes** **No**	23 88	38 91	0.12

In multivariate analyses, it was found that ≥ 3 times antibiotic use increases the risk of infection with MDR bacteria by 4.62 times compared to not using antibiotics. Also, the history of urinary tract infection in the last 6 months has increased the risk of infection with MDR bacteria approximately 2-fold. In addition, being male and being over 65 years increased the risk 3-fold (Table 3).

**Table 3:** Examination of MDR bacterial urinary tract infections by binary logistic regression analysis according to history of AB use, age, and some other characteristics

Features	β	S.E	Wald	p	Exp(β)	95% CI for EXP(B)	
						Lower	Upper
**UTI history** **Absent (Referans)** **Present**	Ref. 0.741	Ref. 0,343	Ref. 4.671	Ref. 0.031	Ref. 2.099	Ref. 1.071	Ref. 4.112
**Not using antibiotic** **Using one time antibiotic** **Using two times antibiotic** **Using three or more times antibiotic**	Ref. -0.161 -0.210 1.530	Ref. 0.365 0.413 0.633	Ref. 0.195 0.258 5.843	Ref. 0.659 0.612 0.016	Ref. 0.851 0.811 4.620	Ref. 0.416 0.361 1.336	Ref. 1.740 1.822 15.980
**Sex** **Female** **Male**	Ref. 1.130	Ref. 0.395	Ref. 8.185	Ref. 0.004	Ref. 3.096	Ref. 1.427	Ref. 6.716
**Age** **<65** **≥65**	Ref. 1.075	Ref. 0.437	Ref. 6.047	Ref. 0.014	Ref. 2.929	Ref. 1.244	Ref. 6.896
**Being ≥65 year man**	-1.611	0.593	7.384	0.007	0.200	0.062	0.638
**Constant**	-0.764	0.286	7.158	0.007	0.466		

## Discussion

MDR bacteria are increasingly causing UTIs. It is very important to know the risk factors for MDR bacteria in CA-UTIs because it is life-saving to start effective antibiotic therapy, especially in urosepsis patients. The present study provided some important insights into the knowledge about risk factors of MDR bacteria in CA-UTIs. History of UTI in the last 6 months, using antibiotics more than 3 times in the last 3 months, being male gender, and being ≥ 65 years old were found as risk factors that increased UTI with MDR bacteria.

Our study showed that frequent antibiotic use is an important risk factor for the development of CA-UTI with MDR bacteria. According to our results, three or more times antibiotic use increases the risk of infection with MDR bacteria by 4.62 times compared to not using antibiotics. Interestingly, one or two times antibiotics usage does not increase the risk. The use of antibiotics such as third-generation cephalosporins, aztreonam, quinolones, trimethoprim-sulfamethoxazole, and aminoglycosides in previous months has been identified as a possible risk factor for MDR in previous studies.9 Unfortunately, Turkey is one of the countries where antibiotics are frequently used. Antibiotic consumption in Turkey was found 42.3 defined daily dose (DDD)/inhabitants per day in 201 and 41.5 DDD/inhabitants per day in 2015.^11^ An indication of the prevalence of MDR bacteria due to the overuse of antibiotics is the fact that most of the community-acquired Gram-negative uropathogens identified in our study were MDR bacteria. Approximately, 54 % of the identified pathogens in this study were MDR.

Being a male gender was found as an independent risk factor for MDR. In a multicenter study from South and Eastern Europe, Turkey and Israel, the male gender was found risk factor for MDR bacteria in complicated UTIs.^12^ In another study, Wang et al.^13^ found that MDR hospital-acquired infections were more common in males. Also, Bischoff et al.^14^ identified that residents in nursing homes, male gender, hospitalization within the last 30 days, renal transplantation, antibiotic treatment within the last 30 days, indwelling urinary catheter and recurrent UTI as risk factors for MDR in patients admitted to an emergency department. In another study in Mexico, being a male gender was found as a risk factor for UTIs with MDR bacteria.^15^ Our study is different from all other studies because patients consist only of CA-UTIs. UTIs seen in men are considered complicated and antibiotics are given for a long time. We think that long-term antibiotic use increases the incidence of MDR bacteria in men.

According to our results, advanced age (≥ 65 years old) was found another risk factor for MDR bacteria. This finding was also found in some other studies. A study from Israel reported that fosfomycin resistance in Gram-negative uropathogens is more often found in elderly patients.^16^ Wang et al.^13^ declared that MDR bacteria were more prevalent in elderly patients (≥ 60 years). In a large study from Scotland that contains 40984 isolates, increasing age was found independent risk factor for MDR.^17^ Due to the weakening of the immune system with aging, treatment of UTIs becomes difficult and recurrency has occurred more common in this group. Another finding of our study supports this thesis. Our results revealed that the history of urinary tract infection in the last 6 months was increased the risk of infection with MDR bacteria approximately 2-fold.

The study has several limitations that could limit the generalizability of our findings to all CA-UTIs. The cases we evaluated in this study consisted only of patients who applied to the hospital and did not include patients who applied to primary health care institutions. Particularly complex and difficult to treat patients presenting to the urology and infectious diseases outpatient clinic will be biased against resistance. The fact that the patients included in our study consisted of complicated and difficult to treat patients who applied to these clinics may have caused a high rate of resistant bacteria that we detected. However, we think that this bias should not affect the relationship between resistance and other variables.

## Conclusion

We demonstrated an increased dose-response relationship between cumulative antibiotic exposure and MDR. MDR bacteria risk was higher in patients using three or more antibiotics in the last three months. Being male, old age and UTI history in the last 6 months were other independent risk factors. Doctors treating CA-UTIs should consider these risk factors when making an antibiotic decision.
